# Management of a rectal entrapped foreign body: a case report

**DOI:** 10.11604/pamj.2024.48.162.43548

**Published:** 2024-08-08

**Authors:** Hiba Ben Hassine, Amina Chaka, Ferdaoues Ouertani, Sadek Ben Jabra, Ibtissem Korbi, Faouzi Noomen

**Affiliations:** 1 Department of Visceral Surgery, Fattouma Bourguiba Hospital, Monastir University, Monastir, Tunisia

**Keywords:** Foreign body, rectum, surgery, case report

## Abstract

Entrapped rectal foreign bodies are being encountered more frequently in clinical practice. They are most often related to sexual behavior or sexual assault. The presence of rectal foreign bodies poses a complex challenge for contemporary surgeons due to multiple factors such as the nature of the object, individual anatomy, duration since insertion, potential accompanying injuries, and degree of local contamination. Managing these cases proves challenging, typically because patients often delay presentation after multiple unsuccessful attempts at self-removal. This study aimed to report the case of a male who presented with an entrapped rectal foreign body related to his sexual behavior. As we could not extract the object with the transanal approach, he was treated by an operative method. Managing patients with rectal foreign bodies presents challenges that require a systematic approach. While most cases can be effectively managed conservatively, surgical intervention may be necessary.

## Introduction

Entrapped rectal foreign bodies are being encountered more frequently in clinical practice. They are most often related to sexual behavior or sexual assault [1]. The presence of rectal foreign bodies poses a complex challenge for contemporary surgeons due to multiple factors such as the nature of the object, individual anatomy, duration since insertion, potential accompanying injuries, and degree of local contamination. Difficulty in diagnosis arises from patients' hesitance to seek medical assistance and reluctance to disclose details about the incident. Managing these cases proves challenging, typically because patients often delay presentation after multiple unsuccessful attempts at self-removal. The management of patients with rectal foreign bodies presents significant challenges. This study aims to report the case of a male patient who presented with an entrapped rectal foreign body. When transanal extraction proved unsuccessful, an operative method was employed for treatment.

## Patient and observation

**Patient information:** a 49-year-old man was received at the surgical emergency department for an obstructive syndrome characterized by vomiting, absence of bowel movements, and gas, as well as diffuse abdominal pain persisting for two days.

**Clinical findings:** the physical examination revealed a hemodynamically stable patient with a tympanic distended abdomen, no guarding, no signs of peritonitis and no palpable mass. On rectal examination, the rectal ampulla was empty with no melena or rectal bleeding.

**Timeline of current episode:** the patient´s medical history showed no history of abdominal surgery or obstructive syndrome. Following an interview, the patient reported that they had not been a victim of sexual abuse. Instead, the patient admitted to engaging in various sexual behaviors involving multiple objects as part of solitary sexual practices.

**Figure 1:**
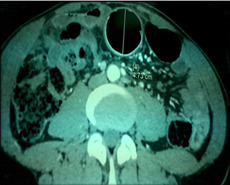
uncommon ectopic placement of foreign body at the rectosigmoid junction: a computerized tomography scan with contrast showed colonic distension upstream of a 10 cm foreign body at the rectosigmoid junction

**Diagnostic assessment:** laboratory investigations revealed the presence of a biological inflammatory syndrome with leukocyte count, 15 x 10^9^/L; neutrophils, 83.3%; and a C-reactive protein (CRP) of 75. An abdominal computerized tomography scan with contrast showed colonic distension upstream of a 10 cm foreign body at the rectosigmoid junction (Figure 1). The patient reported a delayed presentation after multiple unsuccessful attempts at self-removal.

**Diagnosis:** the diagnosis of an entrapped rectal foreign body at the rectosigmoid junction was retained, caused by delayed presentation after multiple unsuccessful attempts at self-removal. Emergency surgery was planned.

**Figure 2:**
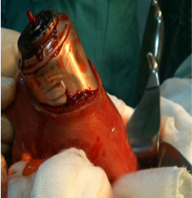
surgical view of the foreign body; intraoperative images showing the foreign body after colotomy

**Therapeutic interventions:** we attempted a trans-anal extraction by two-handed manipulation but without success. Therefore, given the failure of the expulsion, we decided to operate on the patient via the midline, the exploration found a migration of the foreign body to the rectosigmoid junction. A colostomy was performed with extraction of the foreign body followed by simple suturing (Figure 2).

**Follow-up and outcome of interventions:** the postoperative course was simple. He was discharged to home on the fifth postoperative day without complications. The patient was referred to a psychiatric consultation. He remains asymptomatic and in excellent clinical condition.

**Patient perspective:** “I expect to be cured. I continue with treatment and clinical follow-up”.

**Informed consent:** the patient gave written consent for his personal or clinical details along with any identifying images to be published.

## Discussion

Foreign bodies of the rectum (FBR), whether accidental or intentional, are no longer a rare disease in Tunisia. The reason for insertion is not always recognized by the patient, who sometimes alleges an unfortunate accident, as was the case with our patient. In adults, FBR is mostly inserted for sexual purposes [1]. These objects inserted in the rectum are variable, depending on the deviant imagination of the patients [1,2]. They should all be considered potentially dangerous as they can cause significant injury [3]. Indeed, perforation of the recto-sigmoid is reported in 3-17% of cases [4]. They can be spontaneous or secondary to extractions [1,3]. For reasons of modesty or fear of humiliation, patients often do not seek immediate medical attention in the hope that the object will be ejected spontaneously [5]. This delays diagnosis and treatment, thus increasing the risk of complications. In all cases, the clinical presentation is varied, depending on the presence or absence of complications [6]. Thus, patients may be asymptomatic or suffer from constipation, difficulty in evacuating gas, abdominopelvic pain, perianal or anal pain, and rectorragies [1,5]. The physical examination aims to exclude peritonitis; the rectal examination assesses the distance between the foreign body and the anal margin and assesses the competence of the sphincter [3]. X-raying the abdomen without preparation makes it possible to define the nature, size, and shape of the foreign body, and its location, and to exclude pneumoperitoneum [7].

Management of FBR can be difficult because of late consultation after multiple attempts by patients themselves to remove the foreign body. Other factors such as the size, shape, and migration of foreign bodies may also make it difficult to search for and remove them by lower route [1,8]. Thus, depending on their nature and height, several surgical and non-surgical techniques have been described for removing FBR. An attempt to recover the foreign body manually or endoscopically is always justified in the first instance, with or without sedation to allow the anal sphincter to relax. In case of failure, or if there is evidence of significant intestinal injury or even perforation, a laparotomy is justified [9]. In 75% of cases, the foreign body can be removed without surgery [1]. Psychological support and sexual therapy should be suggested to the patients, but in a large series of 30 patients, none wanted such advice [10].

## Conclusion

The diagnosis of foreign bodies in the rectum is generally easy to make based on interrogation, physical examination and X-ray of the abdomen without preparation. The management of patients with rectal foreign bodies presents challenges that require a systematic approach. While most cases can be effectively managed through conservative means, surgical intervention may be necessary in select instances.
